# Sex Ratio Modulates Reproductive Output and Dung Burying Behavior in Dung Beetle *Gymnopleurus sturmi* (Macleay, 1821) (Coleoptera: Scarabaeidae)

**DOI:** 10.1002/ece3.72289

**Published:** 2025-10-16

**Authors:** Alberto Zamprogna, José Serin, Marie‐Ange René, Hasnae Hajji, Patrick Gleeson, Saleta Pérez Vila, Jean‐Pierre Lumaret, Gaylord Desurmont, Valerie Caron

**Affiliations:** ^1^ CSIRO European Laboratory, Campus International de Baillarguet Montferrier‐sur‐Lez France; ^2^ Laboratoire de Biotechnologie, Conservation et Valorisation Des Ressources Naturelles, Faculté des Sciences de Dhar El Mehraz Université Sidi Mohamed Ben Abdellah Fez Morocco; ^3^ Health and Biosecurity, CSIRO Black Mountain Acton Australian Capital Territory Australia; ^4^ European Biological Control Laboratory, USDA, Campus International de Baillarguet Montferrier‐sur‐Lez France; ^5^ National Collections and Marine Infrastructure, CSIRO Black Mountain Acton, Australian Capital Territory Australia

**Keywords:** competition, dung beetle, nesting behavior, Scarabaeidae, sex ratio

## Abstract

Dung beetles are important ecosystem engineers as they play an important role in recycling faces from animals. Dung beetles have evolved different behaviors, including dung ball rolling for their egg and developing offspring. Ball rolling is a complex behavior that varies between species. In some species, males roll the dung ball and females choose partners based on this, while in other species, males and females work together to form the ball. Competition can be fierce with fighting, and ball stealing is common. *Gymnopleurus sturmi* is a ball rolling species that exhibits gregarious behavior with adults congregating on a dung source. This study assesses sex‐related roles in ball rolling as well as the impact of varying sex ratios on the number of balls produced, either left at the surface or buried and fertilized, and emergence rates of the offspring. The theoretical number of offspring per female was used as a measure of fitness. Results show that both males and females can produce dung balls, and higher numbers were obtained when males and females were separated. Female‐biased sex ratio produced mostly buried and fertilized balls, while male‐biased sex ratio produced more unburied balls left on the surface. When females were alone, they produced the maximum number of total dung balls compared to the rest of the treatments. On the other hand, emergence rate was found to be higher when more males were present. When females were alone, emergence rate was extremely low, suggesting reduced sperm storage. Using the theoretical number of offspring per female, no difference in fitness was observed when males and females were both present. In a gregarious species like *G. sturmi,* finding a partner would be easier than for other dung beetle species, which could explain an increasing competition between males and reducing the need to store sperm for the longer term. This study highlights the diversity of behaviors present in this species.

## Graphical abstract

This study examines the sex‐specific roles and the impact of sex ratios on dung ball production and offspring emergence in the dung beetle species *Gymnopleurus sturmi*. It finds that both males and females can produce dung balls, with females producing the most when alone, while male presence increases offspring emergence rates. The research suggests that in this gregarious species, easy access to mates may lead to increased male competition and reduced need for long‐term sperm storage in females.

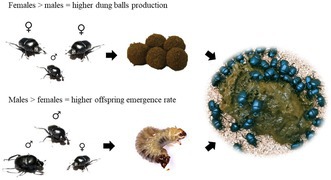

## 1 Introduction

Dung beetles (Coleoptera: Scarabaeidae) are essential to terrestrial ecosystems, providing a series of critical services including aiding soil aeration, nutrient recycling, and overall ecosystem functioning (Beynon et al. 2015; Bornemissza and Williams 1969; Brown et al. 2010; Nichols et al. 2008). They also reduce pest populations associated with dung. For example, they disrupt the breeding cycles of parasitic flies, thereby reducing disease prevalence in cattle (Cessna 2017; Hughes et al. 1978). Since the development of industrial farming in Australia, dung beetles have become increasingly vital (Bornemissza 1960). Local beetle species were not adapted to process the large, moist dung pads from imported livestock, leading to issues such as decreased pasture productivity, fly infestations, and increased parasitism in livestock (Doube 2018). To tackle these challenges, Australia, since the 1960s, has imported dung beetles from Europe and Africa (Edwards 2007). This initiative has proven effective, reducing dung accumulation on pastures, improving soil fertility, and decreasing reliance on chemical fertilizers (Doube 2018). Economically, dung beetles are highly valued in Australian agriculture, saving millions annually in pest control and pasture management expenses (Vieira et al. 2024).

One unique aspect of the Scarabaeinae subfamily is their behavior of shaping dung into balls for egg‐laying and larval development (Hanski and Cambefort 1991; Simmons and Ridsdill‐Smith 2011). Tunneler species bury dung balls in tunnels under the dung source while roller species roll the ball away from the dung source, burying it some distance away. Ball rolling behavior is thought to have evolved to avoid competition quickly and to ensure a source of food for offspring (Byrne et al. 2003). Ball rolling is a complex behavior that varies between species (Halffter et al. 2011). Rollers seem to prefer a straight line as a way of moving away from the dung source, even if obstacles or other impediments are present (Matthews 1963). In some diurnal species, dung beetles seem to follow the position of the sun (Hajji et al. 2023), or other environmental factors (Baird et al. 2010), while a correlation between the positions of certain celestial bodies and the rolling direction of nocturnal species has been demonstrated by Dacke et al. (2004).

Interactions between individuals is paramount in dung rolling species, with positive and negative interactions occurring at every stage of the ball making and rolling process. Cooperative behaviors between males and females have been observed in many species, but are task and species specific. For example, ball forming can be performed by one sex or the other, or both: in *Scarabaeus bohemani* (Harold, 1868), it is the male who makes the ball (Tribe 1976), while in *Kheper nigroaeneus* (Boheman, 1857) males and females make the ball together. There seems to be an advantage in making balls cooperatively as balls made by a pair are larger compared to balls made by a single female or male (Edwards and Aschenborn 1988). Transporting the ball can be done cooperatively as in *Sisyphus* spp. (Tocco et al. 2024), by the male singly with the female in tow as in *Scarabaeus* spp. (Halffter et al. 2011), or with the female hitching a ride on the ball as in *K. nigroaeneus* (Edwards and Aschenborn 1988). Ball burying and nest making is done once an adequate site has been found, and as for ball making and transporting, it can be done singly or cooperatively. In *Scarabaeus catenatus* (Gerstaecker, 1871), both males and females can roll and tunnel for nesting, with females completing the brood nests and males leaving after oviposition (Sato 1997). However, the presence of a male does not translate into an increase in buried ball production and reproductive output (Sato 1998), highlighting the complexity behind the evolution of these behaviors.

Negative interactions between individuals due to fierce intraspecific competition are common in rolling dung beetle species with much fighting and ball stealing, usually between males (Edmonds and Halffter 1982). Ball stealing depends on many factors including the size of the opponent, reproductive status, and the resource available (Chamorro‐Florescano et al. 2011). Lower resource availability and a higher number of males, therefore, increase aggression.

In *Gymnopleurus sturmi*, beetles congregate in large numbers on a dung pad, with single beetles seemingly preferring to join the crowded pad instead of colonizing empty ones nearby (Hajji et al. 2023). Therefore, even when more resources are available, *G. sturmi* remains in groups. Aggregation of individuals could be advantageous as it should be easier to find a partner, but competition is more acute with fighting and ball stealing frequently observed (Hajji et al. 2023), which may potentially lead to different behaviors when ball rolling.

*Gymnopleurus sturmi* is widely distributed in the Mediterranean Basin (Numa et al. 2020). While it is declining in Europe, Morocco and Tunisia still maintain large populations of this species (Haloti et al. 2006; Ruiz 1995). They are mostly found in open and dry sites with clay and sandy soils, where they mainly utilize sheep droppings (Lumaret et al. 2015). Despite its ecological importance, *G. sturmi* remains under‐researched compared to other dung beetle species. In a recent study from Hajji et al. (2023), the direction of ball‐rolling and how this is mediated by the orientation of the sun was investigated, hypothesizing that this strategy is defined by competition avoidance purposes, as also highlighted in other species (Baird et al. 2010). The respective roles of males and females in ball rolling have yet to be investigated for this species, which is particularly interesting in the context of the evolution of its gregarious behavior.

The distribution of the tasks between males and females regarding the genus *Gymnopleurus* was firstly described by Jean‐Henri Fabre (1921), who studied two species in France, *G. mopsus* (Pallas, 1781), and *G. flagellatus* (Fabricius, 1787). He observed that in these species, the female made a ball and rolled it to the place where it was buried, pushing it with her hind legs while her head was turned downwards, and that the male did not help to roll it. However, other studies indicated that in the genus *Gymnopleurus*, the male and female work together, with the male pulling and the female pushing the ball (Prasse 1957; Davis and Scholtz 2020). In *Gymnopleurus geoffroyi* (Fuessly, 1775), the cooperation between a pair reduces the production time by approximately one‐third (Prasse 1957), which was also observed in the field in *G. sturmi* (Hajji et al. 2023). These examples illustrate a behavioral heterogeneity in male–female collaboration in the *Gymnopleurus* genus.

The aim of this study was to investigate the roles of males and females in *G. sturmi* by manipulating the sex ratio, and measuring the production of dung balls, the proportion of unburied dung balls compared to the dung balls buried containing an egg, and the rate of offspring emergence. We hypothesized that:

1. Cooperation between males and females in ball production increases the number of balls produced.
2. Female biased sex ratio reduces brood ball production by reducing cooperation between the sexes.
3. Male competition hinders the number of balls produced as male biased sex ratio increases competition and therefore reduces the number of brood balls produced and buried.
4. Offspring emergence rate wouldn't be affected by sex‐ratio.

## 2 Materials and Methods

### 2.1 Gymnopleurus sturmi

*Gymnopleurus sturmi* is a medium‐sized beetle (10–15 mm long) (Baraud 1992) widely distributed around the Mediterranean basin (Lumaret et al. 2015). Little is known of the biology and ecology of this species. Until recently, males and females could not be differentiated morphologically (Zamprogna et al. 2022). Declines in populations have been observed in Europe, while it remains highly abundant in northern Africa (Errouissi et al. 2004; Hajji et al. 2023; Lumaret et al. 2015), with some sites in Morocco having recently experienced lower populations than previously recorded, most likely due to urbanization (Hajji et al. 2025; Janati‐Idrissi 2000). *Gymnopleurus sturmi* can feed on different dung sources including sheep, cattle, and horse, and even human and dog faces, but it prefers sheep dung (Errouissi et al. 2004; Ruiz 1995). In France, *G. sturmi* is active from spring to early autumn and breeds from late spring to early summer (Lumaret 1990). In Morocco, activity occurs from spring to autumn, with beetles being active earlier at lower altitudes (Hajji et al. 2025). It can withstand high temperatures and can be observed in the open when the soil temperature is above 50°C (Hajji et al. 2023). Under laboratory conditions, breeding occurs when air temperatures are above 30°C (Zamprogna pers. Obs.). It should therefore be considered a summer‐active species (Hajji et al. 2025).

### 2.2 Dung Beetle Rearing

*Gymnopleurus sturmi* was collected in Fez Region, Morocco (34°04′14″ N 5°21′55″ W) and imported to France (permit MONI R76‐2021‐077), where they were reared under laboratory conditions at the CSIRO European Laboratory. Beetles were washed, species identification was confirmed, and sex was determined prior to breeding. Rearing was performed in a containment space dedicated to introduced species. Temperature was set at 30°C and relative humidity at 60%. Light was provided by LEDs (KingPower LED Light—GHK Horticulture) in a 14:10 light:dark cycle.

Beetles were reared in white plastic circular 5 L buckets (17 cm height, 22 cm ⌀) with a meshed lid (mesh size 2 mm) in groups of ten (five pairs). The buckets were filled with sand (type dolomite, sourced in Viols‐le‐Fort, France) and vermiculite (Granutec, size: “superfine”) in a 3:2 proportion. Beetles were fed 3 times a week by providing ¼ cup of thawed cattle dung that had been frozen for at least one month. Dung was collected from pasture at an organic farm in Notre‐Dame‐de‐Londres (43°48′25.2″ N, 3°44′27.6″ E). Old dung was only removed once a week to give dung beetles the choice between older and fresher dung.

Once a week, dung beetles were removed from the container, and brood balls containing eggs were collected by sieving the sandy substrate. Brood balls were placed in lidded plastic containers (25 × 16 × 13 cm) filled with moist vermiculite (1 L:20 L water to vermiculite ratio). The fitted lid had holes to allow ventilation. Brood balls were then placed in a controlled temperature cabinet (Plant Growth Chamber model: FH‐1200, Taiwan Hipoint), set at 24°C and 80% relative humidity. Containers were checked regularly until adult emergence. Newly emerged adults were placed in moist vermiculite at 22°C for 6 weeks and fed as often as necessary. After 6 weeks, matings were observably, and beetles were sexually mature (based on previous observations). Beetles were therefore ready to be used for experimental purposes.

### 2.3 Experiment N° 1

The experiment was conducted in a glasshouse at the CSIRO European Laboratory. Temperatures within the glasshouse fluctuated following the outside environment. The average temperature throughout the experiment was 27.30°C ± 0.03°C with a relative humidity of 45.95% ± 0.12%. The beetles were reared in groups of six individuals, with different male: female ratios: 1:1 (3M3F), 1:0 (6M0F), 0:1 (0M6F), 2:1 (4M2F), and 1:2 (2M4F). This species is usually reared with an equal number of males and females (1:1) in our facilities. Doubling the number of males (2:1) would increase potential interactions between males fighting for females, while doubling the number of females (1:2) would decrease them, which should be translated into a different dung ball production. Removing males or females (0:1 and 0:1) should highlight the main role males and females play in making and burying balls. Each treatment was replicated 10 times, resulting in 60 individuals per treatment, and a total of 300 individuals used. Buckets were sifted weekly to harvest brood balls for six consecutive weeks. The balls collected were divided into two groups: (1) unburied balls left on the surface; (2) pear‐shaped balls that had been buried and in which an egg had been laid. The final sum of both types of balls produced per container was used in the analysis.

### 2.4 Experiment N° 2

A second experiment was conducted to determine whether the sex ratio had an effect on emergence rate and offspring size. The experiment was performed in a controlled environment with an average temperature of 27.3°C ± 3.4°C and an average relative humidity of 62.2% ± 12.3%. The setup was similar to the first experiment except that more beetles were used per rearing container and with different sex ratios: Control 1:1 (4M4F), sex‐ratio 0:1 (0M8F), 3:1 (6M2F), and 1:3 (2M6F). As males alone cannot produce offspring, the male alone treatment was not included in this experiment. There were 8 replicates per treatment. Balls were collected weekly for eight consecutive weeks and counted as in experiment 1. Buried pear‐shaped balls were kept in separate containers for each treatment and incubated in a controlled‐temperature cabinet as described above. To maintain the same density of pear‐shaped brood balls for each treatment, and because some treatments produced more brood balls than others, the number of brood balls incubated each week was equal to that of the least productive treatment. In more productive treatments, brood balls were randomly selected and the rest discarded. A total of 612 brood balls (153 per treatment) were incubated. Containers were checked three times a week. Newly emerged individuals were sexed and weighed prior to feeding using a precision scale (PJ 500c, Precisa Gravimetrics AG). Weight was used as a proxy to assess the quality of the developmental environment as previous manipulations with this species and other dung beetle species have shown a correlation between environmental conditions during development (moisture, dung quality) and adult size and weight, with optimal conditions producing larger and heavier beetles (pers. obs). Emergence dates were also recorded to calculate development time.

### 2.5 Statistical Analyses

All statistical analyses were conducted using R version 4.4.2 (2024‐10‐31 ucrt) – “Pile of Leaves” in the RStudio 2024.12.0 Build 467, “Kousa Dogwood” Release (2009–2024 Posit Software). R Packages used were _dyplr (Wickham et al. 2023), _ggplot2 (Wickham 2016), and _rstatix (Kassambara 2019).

For the two experiments, the impact of sex ratio on the production of balls left at the surface, the number of balls buried with an egg (pear‐shaped), and the total number of balls produced was investigated. In the first experiment, the numbers of unburied balls and buried pear‐shaped balls did not follow a normal distribution, and therefore, a Kruskal–Wallis rank sum test was used with sex ratio as the treatment, followed by pairwise comparisons using the Wilcoxon rank sum test with a continuity correction. As the total number of balls produced followed a normal distribution, a one‐way Analysis of Variance (ANOVA), with subsequent pairwise comparisons conducted via Tukey's Honestly Significant Difference (HSD) test, was performed.

For the second experiment, unburied balls exhibited heterogeneity of variance, as suggested by a failed Levene's test. Consequently, the impact of sex ratio on balls left at the surface was analyzed using the Kruskal–Wallis test, with further pairwise comparisons made using the Wilcoxon rank sum test. The numbers of pear‐shaped balls and the total number of balls produced followed a normal distribution; therefore, a one‐way ANOVA type 2 was performed, followed by a Tukey multiple comparison test to assess the differences between groups. The effect of sex ratio on offspring emergence rates was quantified using Pearson's Chi‐squared tests, with subsequent pairwise analyses employing Pearson's Chi‐squared test with Yates' continuity correction (*α* = 0.05). In addition, the average emergence rate for each treatment was determined and subsequently applied as a fixed coefficient in the following formula: [Number of pears/Number of females]/Emergence rate. This calculation yielded a value referred to as the “theoretical number of offspring per female.” These values have also been analyzed statistically, using the Kruskal–Wallis test and Wilcoxon rank sum test pairwise comparisons, since data were not normally distributed.

The offspring weight at emergence did not conform to a normal distribution, and the effect of sex ratio on weight was thus analyzed using a Kruskal–Wallis rank sum test. Pairwise comparisons were carried out using the Wilcoxon rank sum test with continuity correction, applying Holm's method for *p*‐value adjustment.

## 3 Results

### 3.1 Experiment N°1

**FIGURE 1:**
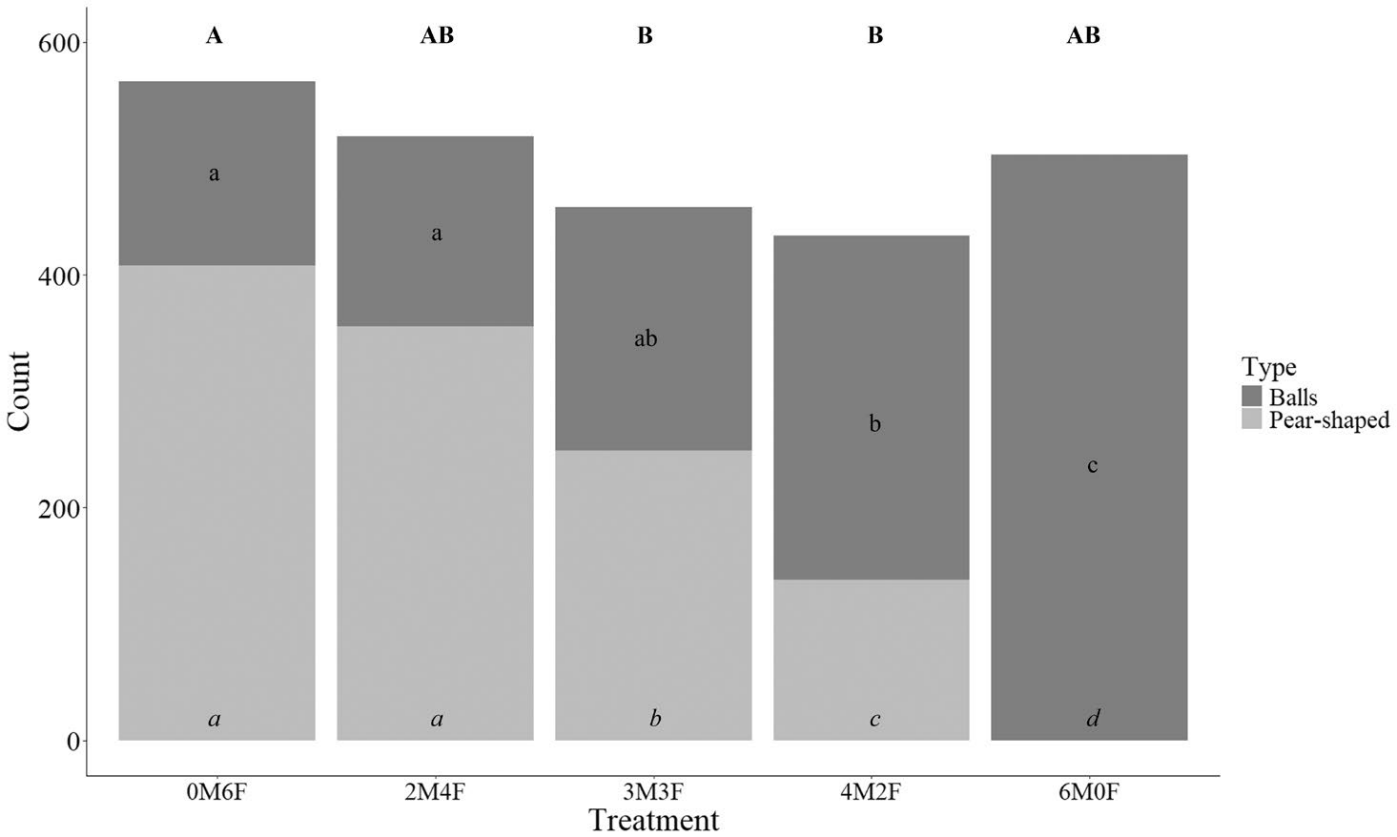
Number of balls left at the surface (balls) and buried balls with egg (pear‐shaped)) produced by *Gymnopleurus sturmi* reared under different sex ratio. M:Male, F:Female. 0M6F = 0 male, 6 females, 2M4F = 2 males, 4 females, 3M3F = 3 males, 3 females, 4M2F = 4 males, 2 females, 6M0F = 6 males, 0 females. Letters indicate statistical significance. Letters in *italic*: Pear‐shaped balls, lower case letters: For balls left at the surface, and CAPITAL LETTERS: Total number of balls produced (sum of balls left at the surface and pear‐shaped balls).

Over the course of this study, 2480 balls were produced, with 1329 unburied balls and 1151 buried pear‐shaped balls, giving a buried‐to‐unburied ball ratio of 0.46. There were significant differences in total balls produced among treatments (*F*_4,45_ = 4.585, *p* = 0.003). The female‐only treatment (0M6F) produced significantly more balls in total than the treatment with equal numbers of males and females (3M3F) and the treatment with a 2:1 sex ratio (4M2F) (Figure 1). Buried pear‐shaped ball production decreased and unburied balls increased with increasing numbers of males. Pear‐shaped ball production across the different treatments revealed significant differences (χ^2^ = 43.039, df = 4, *p* < 0.0001). Notably, no balls were buried in the male‐only treatment (6M0F). All treatments exhibited significantly different levels of buried pear‐shaped ball production, with one exception: the treatments with no males and six females (0M6F) and two males with four females (2M4F) were not significantly different from each other (Figure 1). *p*‐values are presented in Table A1 in the appendix section.

A significant effect of treatment on the number of unburied balls was observed (χ^2^ = 36.564, df = 4, *p* < 0.0001). Specifically, the quantity of balls was positively correlated with the number of males present, with the highest numbers recorded in the male‐only treatment (6M0F). Subsequent pairwise comparisons (Table A1) revealed that the male‐only group (6M0F) differed significantly from all other treatment groups. No significant differences were found in the number of unburied dung balls between treatments with a higher proportion of females (0M6F, 2M4F, 3M3F). However, the 2:1 sex ratio (4M2F) produced significantly more unburied balls than the two highest female counts (0M6F and 2M4F) but not from the 1:1 sex ratio (3M3F).

### 3.2 Experiment N°2

**FIGURE 2:**
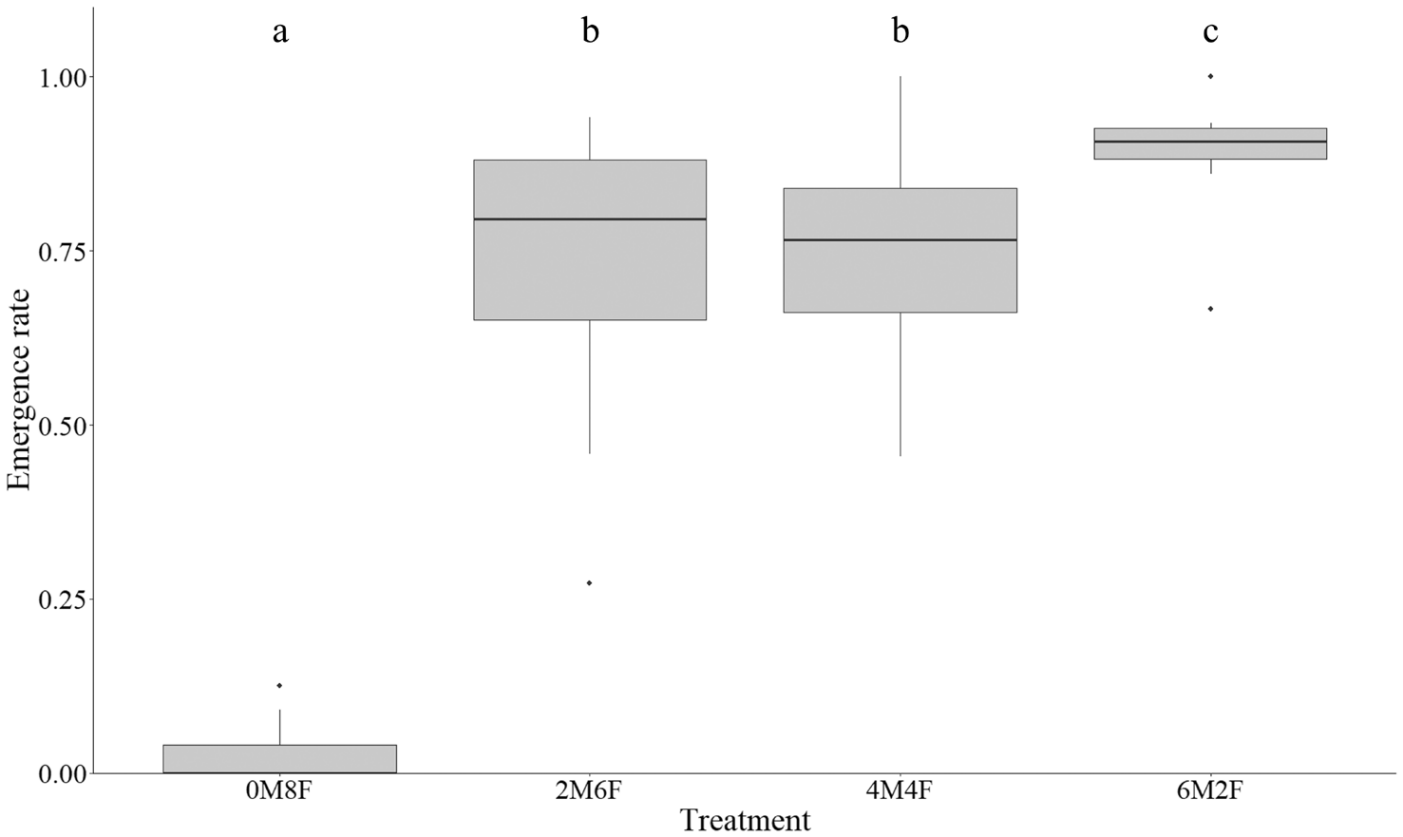
Offspring emergence rate of *Gymnopleurus sturmi* reared with different sex ratio. M:Male, F:Female. 0M6F = 0 male, 6 females, 2M4F = 2 males, 4 females, 3M3F = 3 males, 3 females, 4M2F = 4 males, 2 females, 6M0F = 6 males, 0 females. The horizontal lines inside the boxes represent the medians, the top and bottom edges of the boxes represent 25%–75% quantiles, and whiskers present the range of the data. Outliers are represented by black dots. Letters indicate statistical significance.

**FIGURE 3:**
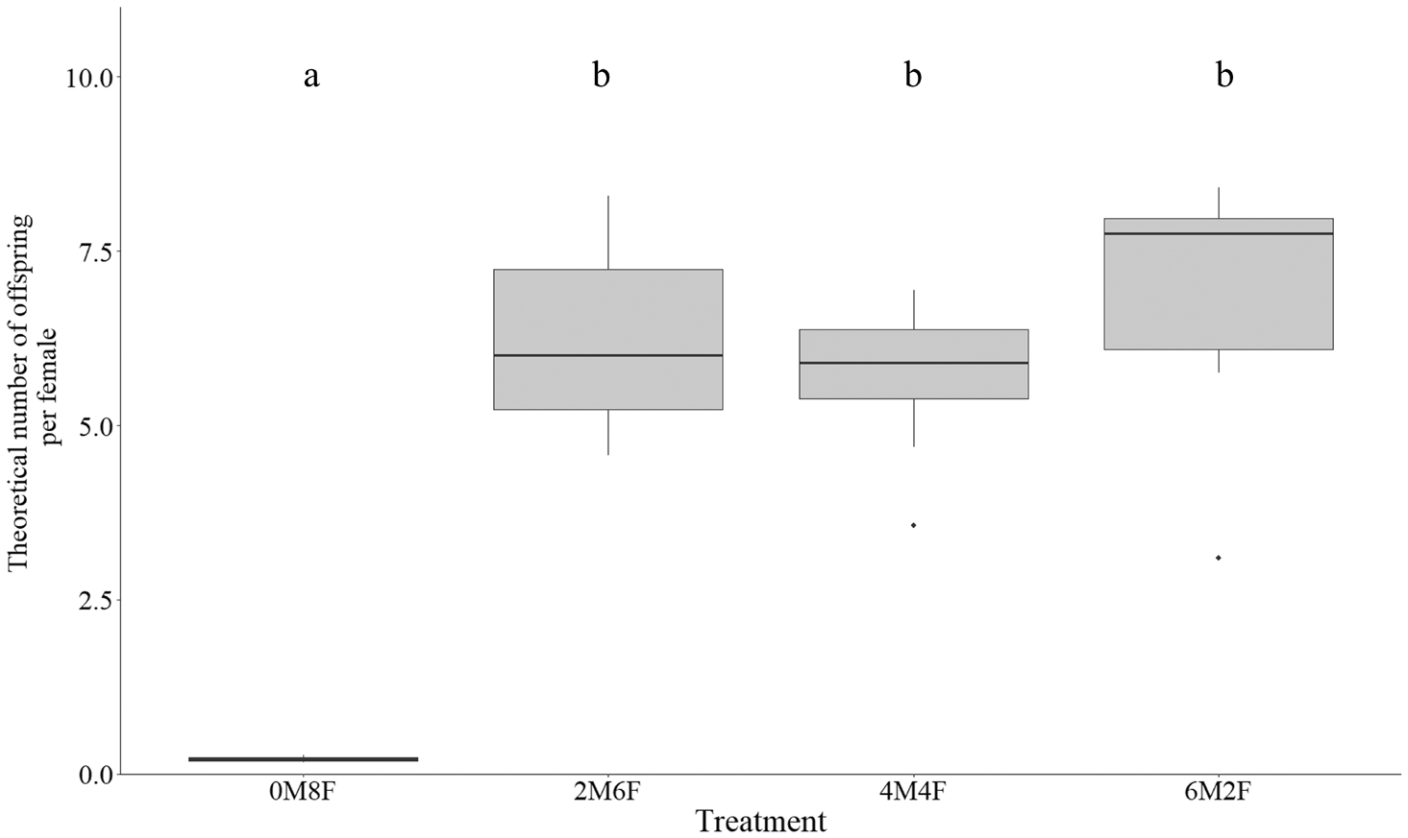
Theoretical number of offspring per female of *Gymnopleurus sturmi* reared under different sex ratio. M:Male, F:Female. 0M6F = 0male, 6 females, 2M4F = 2 males, 4 females, 3M3F = 3 males, 3 females, 4M2F = 4 males, 2 females, 6M0F = 6 males, 0 females. The horizontal lines inside the boxes represent the medians, the top and bottom edges of the boxes represent 25%–75% quantiles, and whiskers present the range of the data. Outliers are represented by black dots. Letters indicate statistical significance.

**FIGURE A1:**
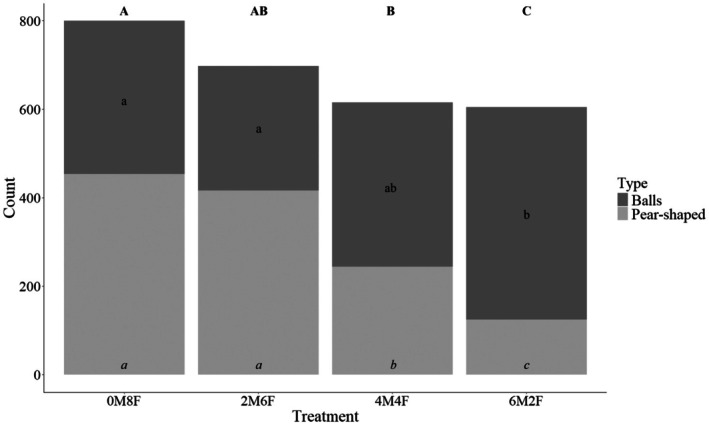
Number of balls left at the surface (balls) and buried balls with eggs (pear‐shaped) produced by *Gymnopleurus sturmi* reared under different sex ratios. M: Male, F: Female. 0M6F = 0 male, 6 females, 2M4F = 2 males, 4 females, 3M3F = 3 males, 3 females, 4M2F = 4 males, 2 females, 6M0F = 6 males, 0 females. Letters indicate statistical significance: *italic*: pear‐shaped balls; lower case letters: balls left at the surface; CAPITAL LETTERS: total number of balls produced (sum of balls left at the surface and pear‐shaped balls). The same significant patterns were observed for both pear‐shaped balls produced and balls left on the surface (for unburied balls and pear‐shaped balls summed: Anova type 2, df = 3, *F* = 40.52, *p* = 2.56e‐10 Anova type 2, for pear‐shaped balls: Anova type 2, df = 4, *F* = 4.585, *p* = 0.00343; for balls; Kruskal–Wallis chi‐squared = 15.246, df = 3, *p*‐value = 0.001618) (Table A2).

As in the first experiment, fewer buried pear‐shaped balls were produced overall than the number of unburied balls, with 1528 and 1762 produced, respectively. The same significant patterns were observed as in Experiment 1 (Figure A1; Table A2). There was a highly significant difference in emergence rates between treatments (*χ* ^2^ = 271.61, *p* < 0.001) (Figure 2). The only‐female treatment (0M8F) had the lowest emergence rate, and this was significantly different from all the other treatments. The treatment with the most males (6M2F) had the highest emergence rate, which was significantly higher than the 1:1 and 1:3 sex ratios 2M6F and 4M4F, which had similar emergence rates (Table A3). The Kruskal–Wallis test shows significant differences in the theoretical number of offspring per female between treatments (*χ* ^2^ = 19.12, df = 3, *p* = 0.0002582). Post hoc Wilcoxon comparisons confirm that treatment 0M8F was distinct from 2M6F, 4M4F, and 6M2F (*p*‐values adjusted to 0.005), while no significant differences were observed between the remaining pairs (Figure 3, Table A4).

On average, newly emerged individuals weighed 0.21 ± 0.01 g. There were significant differences between males and females, with females weighing significantly less than males (Kruskal–Wallis *χ* ^2^ = 4.87, df = 1, *p* = 0.027), with an average weight and standard error of 0.195 ± 0.006 g for females compared to 0.226 ± 0.01 g for males. There was no significant difference in beetle weight at emergence between treatments (Kruskal–Wallis *χ* ^2^ = 4.2375, df = 2, *p*‐value = 0.1202). The time to adult emergence was similar between treatments (Table 1). On average, the first emergence occurred after 37 days and the last after 43 days. Emergence was extremely low for the female‐only treatment (0M8F) compared to the other treatments, with only a 3% emergence rate. The highest emergence rate was with the highest male number (6M2F), with an 87% emergence.

**TABLE 1:** Offspring emergence (mean time to emergence in days ± standard errors, number of male (M) and female (F) emerged, emergence rate) of *Gymnopleurus sturmi* when reared under different sex ratio (0M8F = 0 male, 8 females, 2M6F = 2 males, 6 females, 4M4F = 4 males, 4 females, 6M2F = 6 males, 2 females).

Treatment	Mean first emergence (days)	Mean last emergence (days)	Mean time interval (days)	Emergence			Emergence rate (%)
				M	F	Total	
0M8F	39.7 ± 5	40.3 ± 5	0.7 ± 1	2	3	5	3 ± 2
2M6F	36.8 ± 2	43.6 ± 2	6.9 ± 2	50	62	112	73 ± 8
4M4F	37 ± 2	43 ± 2	7 ± 2	49	62	111	73 ± 6
6M2F	37 ± 2	43 ± 2	7 ± 2	68	63	131	87 ± 3

## 4 Discussion

The aim of this study was to investigate the ball rolling and reproductive behavior of the gregarious dung beetle *G. sturmi*. By manipulating the sex ratio, we investigated the role sex plays in ball rolling and burying, and determined the presence of cooperation between the sexes in this species. The presence of the other sex was not necessary to produce dung balls, as each replicate produced dung balls regardless of the sex ratio. However, the total number of balls varied, as well as the number of buried balls (pear‐shaped) and unburied balls left at the surface. Females can produce dung balls and bury them without males, and males can produce balls without females. However, the types of dung balls were different: the male‐only treatment produced balls that were left on the surface, while the female‐only treatments produced mostly buried pear‐shaped balls. This suggests that the tasks of burying the ball, building the nest, and re‐shaping the ball into a pear are the responsibility of the female and do not require the cooperation of the male.

Surprisingly, the female‐only treatments produced more dung balls overall than the male‐biased sex ratios. It is interesting to note that despite the time‐consuming tasks of burying and reshaping the balls, the female‐only treatment still managed to produce more balls overall. Several reasons could explain these differences. The presence of males could distract the females by forcing them to choose a mate. Dung beetles have complex behaviors when it comes to choosing partners. For example, Berson and Simmons (2018) found that females of the dung beetle *Onthophagus taurus* (Schreber, 1759) prefer males that are vigorously courting and smell different from the average male, while McCullough et al. (2017) highlighted that *O. taurus* females benefit from multiple matings. In the case of a gregarious species, and particularly in a high‐density setup like the one used in the experiments, females may spend more energy interacting with males, and therefore less energy rolling and burying balls. On the other hand, the presence of males also brings competition. Ball stealing and fighting is common in roller species (Halffter et al. 2011; Le Roux et al. 2008; Palmer 1978), and it has been observed in *G. sturmi* with males engaging in multiple fights for ball acquisition with other males (M.‐A. René, unpublished results). Competition between males could have a negative effect and slow the overall ball production.

Both Prasse (1957) and Hajji et al. (2023) observed a shorter production time in ball‐making when cooperation occurred. In our experiment, this would lead to a lower total number of balls produced in male‐only or female‐only treatments, which was not observed. Since ball‐rolling has been identified as a competitive avoidance strategy, and our experiment was conducted in a confined space, the spatial distribution and the consequent density of individuals may have influenced the observed behaviors. In natural settings, Hajji et al. (2023) reported that *G. sturmi* buries dung balls at distances ranging from 3 to 25 m from the dung source, with the presence of obstacles being a determining factor in ball burying behavior. The confined conditions in this study likely increased competition as beetles were unable to disperse beyond their rearing container. Future research should priorities field‐based experiments that incorporate distance and dispersal parameters to better understand these dynamics.

Emergence rates were significantly influenced by sex ratio, with the lowest rates observed in female‐only treatments (0 male, 8 females) and the highest in male‐biased treatments (6 males, 2 females). This suggests that although females could bury balls and produce pears, the presence of males is essential to maximize the number of offspring. A possible explanation would be that mating is required shortly before oviposition, suggesting limited long‐term sperm storage in spermatheca. The aggregative behavior of *G. sturmi* could explain in part the low emergence rate in female‐only treatments. The species tends to aggregate in a single dung pad and form pairs that will proceed to bury the dung ball. After that, both females and males return to the dung source (A.Z., Pers. Observation). The constant presence of new partners with whom to mate and perform the nest‐building activity is thus guaranteed, and therefore, there is no need to efficiently store sperm. Our findings further indicate that egg fertilization occurs during the final stages of dung ball creation. All individuals were reared together for 6 weeks after emergence, and prior to the experiments, they were sexually mature and had opportunities to mate with many partners. If the females had been equipped with spermatheca capable of long‐term sperm storage, there would have been no differences between treatments, as each female would presumably have started the experiments with stored sperm and the ability to fertilize the eggs produced during the experiment. Low sperm storage capacity has been documented in several *Scarabaeus* and *Kheper* dung beetle species (Halffter et al. 2011).

The presence of males may also contribute to higher emergence rates. Little is known about nest construction in *G. sturmi*. It is possible that the male plays a critical role in some part of the nest construction, which helps with the early development of the offspring, leading to higher emergence rates. This would partly follow the conclusion of Favila (1993), who demonstrated the importance of the presence of both parents in the nest to achieve the higher emergence rate.

Finally, increasing emergence rates in the presence of more males could be explained by post‐copulatory mechanisms, such as sperm competition and/or cryptic female choice phenomena (Birkhead and Pizzari 2002). McCullough et al. (2017) studied wild‐caught *O. taurus* females to estimate natural rates of multiple mating: they found them to be highly polyandrous, with 88% of females producing egg clutches fertilized by at least two males. They also showed strong positive correlations between the number of offspring produced and the number of sires, suggesting that females benefit from multiple matings by initiating post‐copulatory mechanisms that favor those males capable of producing more viable offspring to maximize reproductive success. Interestingly, consistent beetle weight at emergence was found across treatments. This suggests that once an egg was fertilized and a pear formed, the quality of the developmental environment was the same regardless of beetle sex ratio and resulted in similarly sized offspring. The theoretical number of offspring per female is a valuable measure for approaching the concept of fitness, as it combines the emergence rate with the offspring produced by each *G. sturmi* female. Based on these data, no significant differences were observed between treatments as long as both sexes were present. When fewer females were present, their lower proportion was compensated by a higher emergence rate. Conversely, when more females were present, they produced a greater number of pears, which compensated for the reduced emergence rate.

## 5 Conclusions

This study shows that cooperation between males and females in *G. sturmi* is essential for successful reproduction. While females can produce dung balls, bury them, and shape them like a pear independently, the presence of males increases emergence rates, suggesting low sperm storage capacity in females or benefits derived from cooperative behavior. While being essential for producing offspring, males in high proportion can hinder the production of buried brood balls containing eggs. Female‐biased sex ratios of 1:2 and 1:3 provided enough males for maximum brood and offspring production. These findings have important implications for understanding reproductive ecology and maintaining optimal sex ratios in laboratory rearing systems. As noted by Thotagamuwa et al. (2023), cooperation between the sexes is a crucial factor to consider in mass‐rearing programs, as it will guide researchers to the appropriate rearing setup that will allow for natural behaviors and lead to successful reproduction. Further research should include behavioral studies of male‐to‐male, female‐to‐female, and male‐to‐female interactions using different sex ratios and densities to investigate aggression, cooperation, and the potential advantages of gregariousness in this species.

## Author Contributions

**Alberto Zamprogna:** conceptualization (lead), data curation (lead), formal analysis (lead), investigation (lead), methodology (lead), writing – original draft (lead). **José Serin:** investigation (supporting), methodology (supporting). **Marie‐Ange René:** investigation (supporting), methodology (supporting). **Hasnae Hajji:** methodology (supporting), writing – review and editing (supporting). **Patrick Gleeson:** conceptualization (supporting), methodology (supporting), writing – review and editing (supporting). **Saleta Pérez Vila:** conceptualization (supporting), methodology (supporting), writing – review and editing (supporting). **Jean‐Pierre Lumaret:** methodology (equal), supervision (equal), writing – review and editing (equal). **Gaylord Desurmont:** formal analysis (equal), supervision (equal), writing – original draft (supporting), writing – review and editing (equal). **Valerie Caron:** conceptualization (lead), formal analysis (lead), methodology (supporting), project administration (lead), supervision (supporting), writing – original draft (supporting), writing – review and editing (lead).

## Conflicts of Interest

The authors declare no conflicts of interest.

## Data Availability

All data used in this study are available in the public repository Dryad Digital Repository and can be accessed via the DOI: https://doi.org/10.5061/dryad.8931zcs2k. The dataset and the script used for the analysis in this study are publicly available.

## 1

**TABLE A1:** Statistical results from *Gymnopleurus sturmi* reared under different sex ratio in Experiment 1 (M: Male, F: Female. 0M6F = 0 male, 6 females, 2M4F = 2 males, 4 females, 3M3F = 3 males, 3 females, 4M2F = 4 males, 2 females, 6M0F = 6 males, 0 females).

Treatments	Total balls produced					Pear‐shaped (buried)					Balls left at surface				
	0M6F	2M4F	3M3F	4M2F	6M0F	0M6F	2M4F	3M3F	4M2F	6M0F	0M6F	2M4F	3M3F	4M2F	6M0F
0M6F	—					—					—				
2M4F	NS *p* = 0.648	—				NS *p* = 0.63	—				NS *p* = 0.732	—			
3M3F	*** *p* = 0.023**	NS *p* = 0.397	—			**** *p* = 0.002**	*** *p* = 0.013**	—			NS *p* = 0.101	NS *p* = 0.101	—		
4M2F	**** *p* = 0.003**	NS *p* = 0.113	NS *p* = 0.955	—		**** *p* = 0.001**	**** *p* = 0.001**	**** *p* = 0.003**	—		**** *p* = 0.003**	**** *p* = 0.003**	NS *p* = 0.055	—	
6M0F	NS *p* = 0.365	NS *p* = 0.990	NS *p* = 0.684	NS *p* = 0.276	—	***** *p* < 0.001**	***** *p* < 0.001**	***** *p* < 0.001**	***** *p* < 0.001**	—	**** *p* = 0.002**	**** *p* = 0.002**	**** *p* = 0.002**	**** *p* = 0.003**	—

*Note:* Results from Wilcoxon post hoc analysis between treatments for unburied balls left at surface and buried balls with eggs (pear‐shaped), and Tukey's honestly significant difference post hoc comparisons for total balls produced (sum of balls left at the surface and pear shaped balls), NS: Non‐significant. **p* < 0.05, ***p* < 0.01 ****p* < 0.001.

**TABLE A2:** Statistical results for *Gymnopleurus sturmi* reared under different sex ratios in Experiment 2 (M: Male, F: Female. 0M8F = 0 male, 8 females, 2M6F = 2 males, 6 females, 4M4F = 4 males, 4 females, 6M2F = 6 males, 2 females).

Treatments	Total balls produced				Pear‐shaped(balls buried)				Balls left at surface			
	0M8F	2M6F	4M4F	6M2F	0M8F	2M6F	4M4F	6M2F	0M8F	2M6F	4M4F	6M2F
0M8F	—				—				—			
2M6F	NS *p* = 0.701	—			NS *p* = 0.701	—			NS *p* = 0.376	—		
4M4F	***** *p* < 0.001**	NS *p* = 0.0001	—		***** *p* < 0.001**	***** *p* < 0.001**	—		NS *p* = 0.599	NS *p* = 0.123	—	
6M2F	***** *p* < 0.001**	***** *p* < 0.001**	**** *p* = 0.007**	—	***** *p* < 0.001**	***** *p* < 0.001**	**** *p* = 0.0077**	—	*** *p* = 0.049**	****** ***p* = 0.05**	NS *p* = 0.123	—

*Note:* Results from Wilcoxon post hoc analysis between treatments for balls left at the surface and Tukey multiple comparison post hoc analysis between treatments for buried balls with eggs (pear‐shaped) and total balls produced (sum of balls left at the surface and pear‐shaped balls) by, NS: Non‐significant. **p* < 0.05, ***p* < 0.01, ****p* < 0.001.

**TABLE A3:** Chi‐squared pairwise comparison for emergence rate of *Gymnopleurus sturmi* reared under different sex ratios M: Male, F: Female. 0M8F = 0 males, 8 females, 2M6F = 2 males, 6 females, 4M4F = 4 males, 4 females, 6M2F = 6 males, 2 females.

Treatment	0M8F	2M6F	4M4F	6M2F
0M8F	—			
2M6F	******* **χ** ^**2**^ **= 155.48 *p* < 0.001**	—		
4M4F	******* **χ** ^**2**^ **= 155.48 *p* < 0.001**	NS χ^2^ = 0 *p* = 1	—	
6M2F	******* **χ** ^**2**^ **= 212.88 *p* < 0.001**	****** **χ** ^**2**^ **= 8.19 *p* = 0.004**	****** **χ** ^**2**^ **= 8.19 *p* = 0.004**	—

*Note:* NS: Non‐significant. **p* < 0.05, ***p* < 0.01 ****p* < 0.001.

**TABLE A4:** Wilcoxon post hoc analysis between theoretical number of offspring produced per female when *Gymnopleurus sturmi* are reared under different sex ratios (M: Male, F: Female. 0M8F = 0 males, 8 females; 2M6F = 2 males, 6 females; 4M4F = 4 males, 4 females; 6M2F = 6 males, 2 females).

Treatment	0M8F	2M6F	4M4F	6M2F
0M8F	—			
2M6F	****** ***p* = 0 9.31e‐4**	—		
4M4F	****** ***p* = 9.23e‐4**	N.S.	—	
6M2F	****** ***p* = 8.99e‐4**	N.S.	N.S.	—

*Note:* NS: Non‐significant. **p* < 0.05, ***p* < 0.01 ****p* < 0.001.
